# Time for a simulation strategy?

**DOI:** 10.1002/jmrs.685

**Published:** 2023-05-10

**Authors:** Naomi Shiner

**Affiliations:** ^1^ Keele University Keele UK

## Abstract

This editorial evaluates the role of virtual reality alongside traditional simulation learning environments for radiography education. With such rapid uptake in recent years, is it time to consider how simulation can be implemented more strategically?

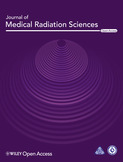

## Introduction

Clinical environments have been challenging for undergraduate students and qualifying radiographers to navigate for several decades.^1^, ^2^ Contributing factors include working with unwell service users, building good relationships with supervisors and patients, and ‘the unknown’.^2^, ^3^ Simulation‐based education (SBE) is now an accepted pedagogical approach to support the transition to clinical practice.^4^, ^5^ Bridge et al.^6^ highlight various resources used in SBE; for this editorial, these are simplified into two broad categories, ‘traditional simulation’ using physical spaces, people, and objects, and ‘Virtual Reality’ (VR).

VR now offers students experiences in different imaging modalities, as outlined by Taylor et al.^7^ and Rowe et al.^8^ in this issue. Acceptance and successful outcomes have led studies to explore the use of SBE as an opportunity to expand placement provision and strengthen cohort sizes.^9^, ^10^ The pandemic has pushed radiography education into a new era, with SBE having a leading role. However, anecdotal evidence suggests many educators have integrated it sporadically into the curriculum due to either necessity (COVID‐19) or simply trialling a new pedagogical approach. It may be time to take a strategic approach to implement both traditional simulation and VR into a radiography curriculum, to maximise investments and promote successful student learning outcomes.

## Comparative Review of Traditional Simulation and Virtual Reality

SBE is no longer limited to traditional environments such as clinical wards and X‐ray suites. Taylor et al.^7^ and Rowe et al.^8^ identify some advantages that VR offers radiography training, including experiencing environments that are more challenging to access, for example, computed tomography (CT); providing a ‘trial and error’ teaching environment, and flexible learning for larger cohorts.^7^, ^8^ Taylor et al.^7^ highlight the relative infancy of this technology in health education compared to traditional simulation that has developed over centuries. Educators should consider how it is integrated into the curriculum, with tangible benefits linked to learning outcomes.^8^ A dominance of analysing student confidence levels is identified by Taylor et al.,^7^ yet confidence does not mean competence. VR currently has a novelty factor, with the potential for reduced student engagement over time.^7^ Longitudinal evaluations will be necessary to understand the potential for the long‐term impact of these investments.^3^

Rowe et al.^8^ provide striking evidence to suggest that training with VR software (Virtual medical coaching) produces a more efficient student, undertaking fewer errors and producing a diagnostic image in less time, compared to students trained using traditional simulation. Furthermore, statistical evidence is provided by Taylor et al.,^7^ suggesting there is a place for VR to aid technical knowledge and decision‐making in CT, with no statistical difference found between using VR and physically operating a real scanner. The similarity in outcomes may mean VR is a feasible alternative for higher education institutes (HEIs) over purchasing real CT scanners for training.^7^ These statistics present a case for displacing traditional simulation in certain situations. Arguably the use of VR is a new pedagogical resource for many educators. Taylor et al.,^7^ highlighted the need for students regularly use of VR, to maintain their knowledge and skills, and perhaps most significantly to receive improved feedback from competent facilitators. The debriefing stage for any simulation activity being that VR or traditional, is heavily evidenced as where learning and reflective practice takes place.^3^

The focus of VR in radiography is centred on image acquisition, with little mention of building relationships with the patient.^7^, ^8^ In an age where personalised‐centred care has become a strong focus of radiography training,^11^ learning communication skills and building relationships is also crucial. Rowe et al.^8^ have taken the bold step to solely use VR to train students in the first year of training, replacing their use of traditional simulation. An alternative strategy has been a blended approach, offering an opportunity for students to practice balancing their technical skills alongside communicating with patients.^9^, ^10^ Ketterer et al.^9^ identified statistically significant improvements in the students' ability to communicate with patients on clinical placement, following simulations using a virtual environment for radiotherapy training, and simulated patients in a radiotherapy setting. A strategy should consider the balance of various learning objectives delivered through simulation.

Traditional simulation is not perfect; participants can suffer anxiety when role‐playing in front of peers.^10^ In contrast, students have reported learning alongside peers, and the ability to see others' reactions provides emotional comfort.^12^ Opportunities to experience unnerving high‐stakes scenarios with peers builds support, which continues into clinical practice.^3^ Perhaps this is where a divide is seen between what traditional simulation and VR can offer, or perhaps is dependent on the scenarios and software design. High‐stakes scenarios are less reported within VR research. It might be argued that working in a VR environment is a more solitary experience or ‘free from distractions’ as Rowe et al. highlights.^8^ Could this reduce the ability to build relationships with others? Interestingly, students have still reported feeling empathy for patients when using Magnetic Resonance Imaging (MRI) VR desktop software (The Institute for Advanced Clinical Imaging) whilst listening to the integrated noise of the MRI sequences.^10^ Taylor et al.^7^ touches on some issues associated with VR, including lack of human interaction. Furthermore, VR or cybersickness could present a problem, prohibiting the user from completing a scenario without discomfort. Hybrid simulation may overcome VR limitations associated with learning objectives linked to non‐technical skills, such as building relationships.

A significant contributing factor altering human emotion is ‘the unknown’.^3^ Shiner^3^ found student engagement within a scenario altered due to fear. When presented with simulated patients suffering open wounds (simulated using moulage) and in pain, students shifted into observational roles, limiting their communication with the patient in fear of making the situation worse. However, on unpacking this issue in the debriefing, students reported a wish to ‘step up, not step back’, recognising this is a situation frequently experienced in clinical practice. It is essential for the transition to practice to provide opportunities to explore personal emotions, as the affective domain can derail the cognitive and psychomotor domains. A simulation strategy should provide opportunities to explore all three domains.

The parity of student experience can be challenging to guarantee solely using clinical practice. Rowe et al.^8^ highlight the parity provided by using software by Virtual Medical Coaching software. Furthermore, the standardisation of simulated practice across HEIs is being explored. However, this would require HEIs or clinical placements to have the same resources to support such a project. Evidence indicates a wide variation of resources on an international scale.^6^ VR offers an opportunity to standardise experiences when imaging pressures within a department reduces capacity for learning. In contrast, there is a requirement that qualifying radiographers are prepared to work under such pressures, meaning exposure with high‐stakes simulation may offer some preparation for qualification. Simulation plays an important role in understanding human factors and should be integrated beyond undergraduate education, to further support transitioning radiographers.

## Theory and Implementation of SBE

The use of simulation is underpinned by theory.^3^ Considering experiential theory, simulation offers an opportunity to reach mastery in a skill, technique, or various scenarios. However, mastery does not come with one‐off simulations. It is important to consider this, when planning the frequency of simulation in the curriculum. VR offers a repeated opportunity for students to practice, whereas traditional simulation is more time and staff intensive, which can limit its use.^6^ Furthermore, considering cognitive load theory, educators should ensure a scaffolded approach to learning is still used, offering students a reduced‐risk environment to build from prior to clinical practice. Browne and Philips^13^ offer a 5‐stage scaffolded approach to integrating simulation into healthcare curricula: (1) online learning; (2) facilitated practical (task training); (3) simulation consolidation (facilitated simulation); (4) simulation days (remote facilitation); (5) clinical practice. Integrating SBE early in the curricula allows students' confidence and competence to build along with the complexity of scenarios.

Alternative frameworks and models are provided which could be integrated into this 5‐stage model offering greater strategic guidance. Taylor et al.^7^ have provided a conceptual framework for VR use, focusing on learning paradigms, reminding educators of the need for continuous refinement. Other theoretical models such as Hyde and Hardy's^11^ theoretical model for delivering patient‐centred care could be used to inform aspects of simulation scenarios, ensuring educators meet the needs of the patients. A simulation strategy allowing both human interaction as well as developing technical skills is paramount.

## Conclusion

This editorial may have raised several questions. What should be taught using simulation and what should not? Where and how much should a HEI invest in resources? Answers to these questions will vary from institute to institute. A strategy should be led by a vision, be informed by audits, student feedback, placement provision, evidenced‐based practice, regulatory and societal bodies' guidance and much more. Following the relatively rapid introduction of simulation to support education during the pandemic, it is time to pause, reflect, and strategise.

## Data Availability

Data sharing not applicable to this article as no datasets were generated or analysed during the current study.
